# The effect of in-office bleaching agents on the Vickers hardness and surface topography of polished and unpolished CAD/CAM composite materials

**DOI:** 10.1038/s41598-023-42415-4

**Published:** 2023-09-15

**Authors:** Rasha A. Alamoush, Jiawei Yang, Abdulaziz Alhotan, Julfikar Haider, Jukka P. Matinlinna, Alaaeldin Elraggal

**Affiliations:** 1 Department of Fixed and Removable Prosthodontics, School of Dentistry, The University of Jordan, Amman, 11942 Jordan; 2 Department of Prosthodontics, Shanghai Ninth People’s Hospital, College of Stomatology, Shanghai Jiao Tong University School of Medicine, Shanghai, China; 3 National Clinical Research Center for Oral Diseases, College of Stomatology, Shanghai Jiao Tong University School of Medicine, Shanghai, China; 4 Department of Dental Health, College of Applied Medical Sciences, King Saud University, 11545 Riyadh, Saudi Arabia; 5 Department of Engineering, Manchester Metropolitan University, Manchester, M1 5GD UK; 6 Division of Dentistry, School of Medical Sciences, The University of Manchester, Manchester, M13 9PL UK; 7 Operative Dentistry, Conservative Dentistry Department, Faculty of Dentistry, Alexandria University, Alexandria, Egypt

**Keywords:** Health care, Health occupations, Medical research, Materials science, Physics

## Abstract

In-office bleaching, using hydrogen peroxide, is effective to remove dental enamel stains. However, bleaching agents can deteriorate surface properties of CAD-CAM materials. This in vitro study aimed to investigate the effect of in-office bleaching agents on Vickers hardness and surface topography of polished and unpolished dental CAD-CAM composite materials (Grandio blocs, Lava Ultimate, BRILLIANT Crios, Cerasmart), and a polymer-infiltrated ceramic network block (Vita Enamic). The specimens were randomly divided into two groups: unpolished or polished. The micro-hardness and surface topography of each group were measured before bleaching, after a 60 min bleaching period, and 24-h and one-month post-bleaching. In-office bleaching significantly influenced the Vickers hardness of both the polished and unpolished CAD/CAM composite blocks, with Vita Enamic exhibiting the least hardness stability among all groups. Furthermore, in-office bleaching significantly influenced the surface roughness of unpolished CAD/CAM composite blocks. There was a significant difference in hardness reduction between the polished and unpolished specimens for most of the investigated materials at different time points. The bleaching did not influence the surface roughness of the investigated polished group, except for Vita Enamic and Lava Ultimate. However, it did influence the surface roughness of the investigated materials in the unpolished group.

## Introduction

Aesthetic CAD/CAM restorative materials in dentistry include predominantly ceramics and resin-based composites^1^. Ceramics are favoured because they offer superior biocompatibility, aesthetics and strength compared and contrasted to resin-based composites^2,3^. CAD/CAM composites have improved mechanical properties compared to the direct resin composites due to the innovative composition and polymerisation modes under high temperature and/or pressure^4,5^. Compared to ceramics, the CAD/CAM composite blocks have similar hardness and stiffness to that of the tooth structure which is beneficial for repair, wear reduction, and, improvement of clinical performance, longevity and machinability of the material^3,6^. Currently, CAD/CAM composite blocks can be classified based on their microstructure as follows: the polymer-infiltrated ceramic network (PICN), which can be described as a porous ceramic network infiltrated with a polymer network, or a resin-composite block (RCB), which is a resin-composite formed by mixing the polymer and filler components under high pressure and high temperature^7,8^. One of the main aesthetic disadvantages of CAD/CAM composite blocks is their susceptibility to staining^9,10^. However, they offer more colour stability than the conventional direct and indirect resin-composite (photo polymerised in the clinic or dental laboratory) materials^11,12^ due to the higher degree of polymerization and improved mechanical properties^13^. Consequently, bleaching of such restorations might be requested after a short- or long-term period to enhance and restore their aesthetic appearance^14^.

The colour stability of dental restorations is very essential for both the patients and clinicians, mainly because of aesthetical reasons. However, it can be affected surprisingly by many intrinsic and extrinsic factors including dietary habits, medical history of the patient, exposure time, and concentration of the staining agents^15,16^. Discolouration of the dental restorations can be influenced by their composition (photo-initiators, activators, resin matrix, silane coupling agent and fillers), physicochemical reactions, hydrophilicity/hydrophobicity, and the water sorption of the materials^17^. Other key factors related to the success of resin composite restorations include incomplete polymerisation, curing time and devices, porosities, and oxygen inhibition at the surface, and surface treatments^18^.

Chemical bleaching methods mainly include carbamide and/or hydrogen peroxide gels in different concentrations of the reactive ingredient. In-office bleaching using hydrogen peroxide is an effective method of removing tooth stains of intrinsic and extrinsic origin^19^. This approach utilises a high-concentration hydrogen peroxide (H_2_O_2_, HP; 35% to 40%) that oxidises the colour stains and pigments on the tooth surface^20,21^. The main advantage of in-office bleaching is the ability to achieve tooth whitening in one dental visit. In contrast, it can cause tooth sensitivity and tissue irritation^22^. Carbamide peroxide (CP) at different concentrations (10% to 20%) is mainly used for home bleaching^23^. The main advantages of home bleaching are ease of use, less chair-side time, and less tooth sensitivity and gingival irritation after bleaching^24,25^. However, such bleaching agents might not be as safe for restorative materials as they are for the tooth enamel, and their use could lead to surface degradation, changes in surface roughness, erosion and ultimately failure^26–28^. Bleaching with hydrogen peroxide, for instance, has been found to affect the three-dimensional polymer network in polymerised composites^14^. Furthermore, in-office and home bleaching agents might increase the staining susceptibility of restorative materials^25,29^.

The surface roughness of the CAD/CAM composite restoration might increase over time^30^ due to the consumption of different food and beverages^9,31,32^ or as a result of teeth brushing^9,33,34^. Increased surface roughness might render the restorative materials more prone to discolouration^32–36^. Furthermore, this might make the restoration more susceptible to bleaching agents and lead to inferior mechanical properties, such as reduced hardness^35^. Many studies have investigated the effect of bleaching agents on colour changes, optical properties, and topography^28,29,36–39^. However, few have investigated the effect bleaching has on the mechanical properties, such as hardness and surface roughness, of both polished and unpolished restorative materials.

This current study aimed to investigate the influence of an in vitro bleaching system (in-office bleaching system) on the Vickers hardness and surface roughness of five CAD/CAM composite blocks. The null hypotheses were as follows: (i) the bleaching agents have no influence on the micro-hardness of the investigated materials; (ii) bleaching duration and material type do not have an influence on the hardness reduction of the investigated materials; (iii) treating the surface of the investigated materials (polished and unpolished) does not impact the hardness reduction after bleaching; (iv) filler weight does not have an impact on the hardness reduction of the investigated materials; and (v) bleaching agents do not have an impact on the surface roughness of the investigated materials.

## Materials and methods

### Sample preparation

Five CAD/CAM blocks were investigated: four resin-composite blocks (RCB), Grandio blocs (Gr), Lava Ultimate (Lu), BRILLIANT Crios (Bc) and Cerasmart (Cs); and one polymer-infiltrated ceramic network (PICN) block Enamic (En), as shown in Table 1. They were selected due to their wider applications as representative CAD/CAM blocks for aesthetic restorations.

**Table 1:** The manufacturers’ compositional information and experimentally determined filler weight percentage^63^ of the materials investigated, and bleaching agent used.

Materials (code)		Manufacturer	Composition by weight (%) represented by manufacturers		Filler composition by weight (%) determined by ashing (SD)
			Polymer matrix	Filler matrix	
Polymer infiltrated ceramic network (PICN)	Vita Enamic (EN)	Vita Zahnfabrik, Bad Säckingen, Germany	14% UDMA + TEGDMA	86% fine structure feldspar ceramic	85.1 (0.1)
Resin composite CAD CAM blocks	Grandio Blocs (GR)	VOCO GmbH, Cuxhaven, Germany	14% UDMA + DMA	86% nanohybrid fillers	84.6 (0.01)
	Lava Ultimate (LU)	3M ESPE, St Paul, MN, USA	20% (bis-GMA, UDMA, bis-EMA, TEGDMA)	80% silica and zirconia nano particles	74.8 (0.1)
	BRILLIANT Crios (BC)	COLTENE, Altstätten, Switzerland	Cross-linked methacrylates (bis-GMA, bis-EMA, TEGDMA)	70% of glass and amorphous silica	70.1 (0.05)
	Cerasmart (CS)	GC Corporation, Tokyo, Japan	Bis-MEPP, UDMA, DMA	71% silica and barium glass nanoparticles	66.1 (0.2)
Bleaching agent	Opalescence Boost	Manufacturer	Type	Composition	
		Ultradent Products, South Jordan, UT, USA	Chemically activated in-office bleaching agent	40% hydrogen peroxide (H_2_O_2_), potassium nitrate and fluoride	

**Figure 1:**
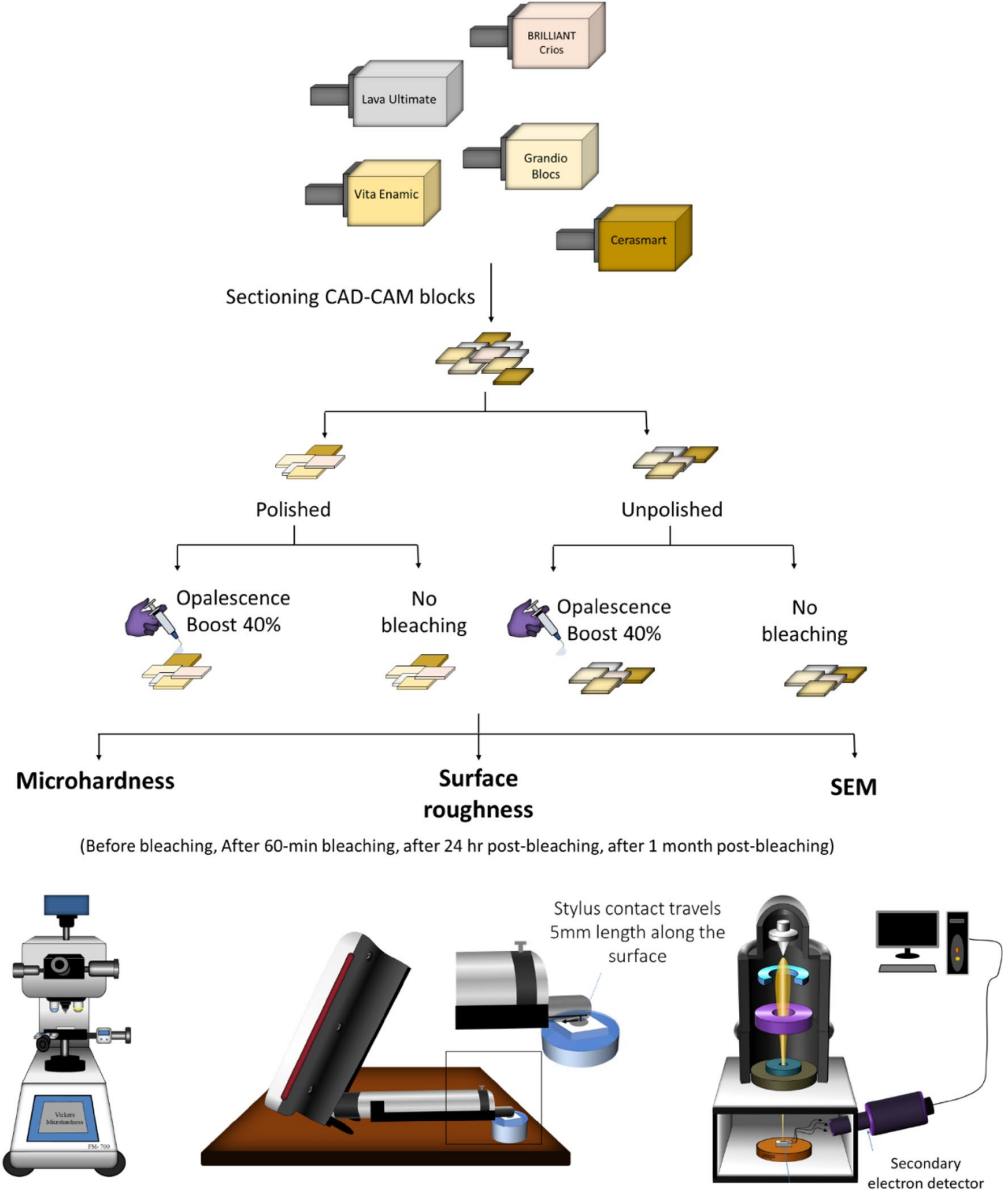
A diagram of overall experimental and characterization procedure.

The CAD/CAM blocks were sectioned into rectangular bar shaped specimens (14 mm × 12 mm × 2 mm) using a cutting machine (MECATCH234, PRESI, France) under constant water cooling (Fig. 1). The specimens were wet-ground and polished using a polishing machine (BETA-VECTOR, Buehler, IL, USA) with silicon carbide (SiC) papers (P1200, P2500 and P4000 grit, Buehler, IL, USA) under water cooling, followed by a 0.25 µm diamond suspension polishing (Meta Di Supreme, Buehler, IL, USA). The specimens were cleansed in an ultrasonic water bath (Ultrasonic Cleaning System; L&R, NJ, USA) for 5 min. The specimen dimensions were confirmed to an accuracy of ± 0.1 mm using a digital calliper. Any specimens not within this range were discarded. The sample size for each experiment was calculated initially using mean differences, standard deviations, and confidence interval of 95% and found to be sufficient with significance level of 0.05.

### Bleaching procedure

Thirty samples of each material (15 polished and 15 unpolished) were randomly distributed into three study groups: four samples were used as control (unbleached), four for Vickers hardness, four samples for surface roughness, and three for SEM analysis. All samples were stored in distilled water at 37 °C for 3 months^40^ (ISO 10993-13, 2010) before the bleaching procedure was performed using Opalescence Boost (in-office bleaching) for three intervals of 20 min (total 60 min). The bleaching procedure was performed by one investigator at room temperature, with the bleaching agent covering the whole top surface of each specimen. At the end of the respective bleaching procedure, the specimens were rinsed with a high-pressure water flow, dried with blotting paper and airflow, and placed in fresh distilled water for subsequent measurements.

### Micro-hardness measurement

Micro-hardness of each specimen (n = 4) was measured using a Vickers micro-hardness instrument (HXD-1000 TMC, Shanghai Taiming Optical instrument, Shanghai, China). A fixed load of 300 gf was applied for 20 s. Ten indentations at a 0.5 mm interval were made randomly on each sample. The machine then automatically calculated the corresponding hardness value and presented the Vickers hardness number (VHN). For in-office bleaching, surface micro-hardness was measured before bleaching, after 60 min of bleaching and at 24-h and 1-month post-bleaching. VHN was also measured for the corresponding control samples from each group. The samples were cleaned and dried before the micro-hardness measurement.

The hardness reduction (HR as a percentage) after bleaching storage was calculated using Eq. (1).

1 $$ {\text{HR}}\% = \frac{{{\text{VHN(before)}} - {\text{VHN(after)}}}}{{\text{VHN(before)}}} \times 100\% $$

where VHN(_before_) and VHN(_after_) indicate the Vickers hardness numbers before and after bleaching respectively for each time point.

### Surface roughness

The surface roughness of each specimen (n = 4) was measured using a stylus contact type profilometer (Mahr Perthometer M1 [L_t_ = 5.6 mm, λ_c_ = 0.800 mm]). R_a_, the arithmetic mean height of the roughness profile, was recorded during the measurement. Three vertical and three horizontal lines were measured on the specimen surface to record six R_a_ values for each specimen, and the mean R_a_ result was subsequently calculated. Surface roughness was also measured before bleaching and at the same timeframes after bleaching and post-bleaching similar to hardness measurement.

### Scanning electron microscope imaging

Each specimen was attached to aluminium stubs (12 mm thickness) with double-sided adhesive tabs. The specimens were sputter-coated with gold and examined with a scanning electron microscope (FESEM, S-4800, Hitachi, Tokyo, Japan) (n = 3). SEM micrographs were produced at × 40k magnifications of representative areas of the samples.

### Statistical analyses

The data were analysed using a statistical software package (GraphPad Prism, version 9.1.2 (226) and found to exhibit normal distribution according to the Shapiro–Wilk test. Two-way ANOVA was performed to investigate the material’s effect, storage time effect, and their interaction. One-way ANOVA was followed by the Tukey’s post hoc analysis for multiple comparisons between different materials and different time points. The Pearson correlation was determined to identify the correlation between the hardness reduction and filler weight percentages, in both the polished and unpolished groups. An independent t-test was performed to measure differences between the polished and unpolished groups (without bleaching, after 60 min of bleaching, 24 h and one-month post-bleaching) and for differences between the bleached and unbleached groups at each time point for each material. All tests were carried out at α = 0.05.

## Results

**Figure 2:**
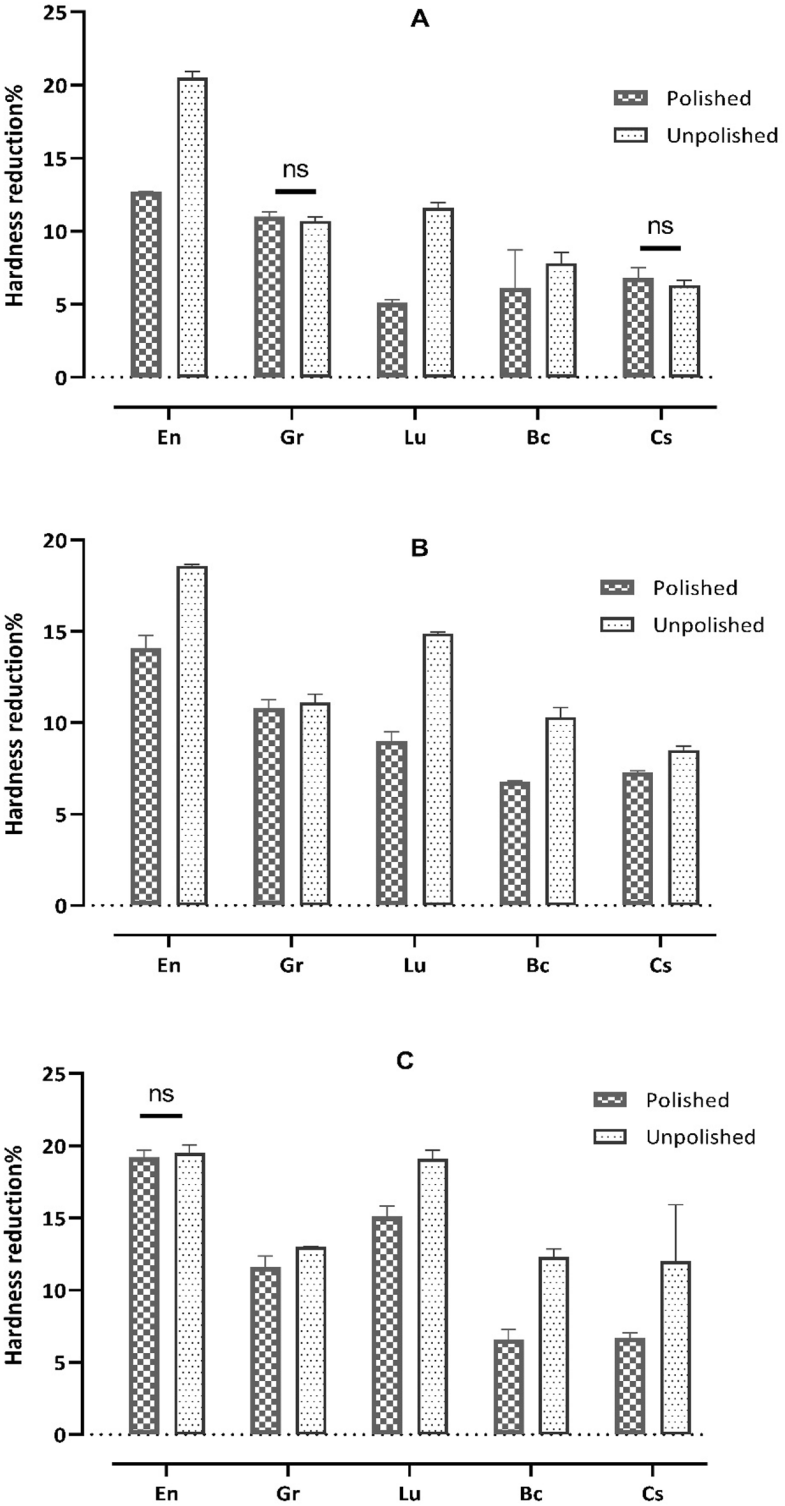
A bar chart illustrating the mean values of hardness reduction percentage (HR %) (**A**) after 60 min, (**B**) 24 h post-bleaching, and (**C**) one-month post-bleaching. Error bars represent the standard deviation and ns indicates ‘not significant’.

The reduction of hardness percentage was calculated 60 min after bleaching, after 24 h and after one-month and for the control unbleached samples at the same time points. There was a significant difference between the bleached and unbleached (control) specimens for each material, as confirmed by the independent sample t-test (Table 2 and Fig. 2).

**Table 2:** The mean and standard deviation values of the reduction of hardness percentage (HR %) of polished and unpolished samples after 60 min in-office bleaching, 24 h and one-month post-bleaching.

Materials		%Reduction of hardness (polished samples); mean (SD)			%Reduction of hardness (unpolished samples); mean (SD)		
		After 60 min bleaching	After 24 h post-bleaching	After 1-month post-bleaching	After 60 min bleaching	After 24 h post-bleaching	After 1-month post-bleaching
PICN	En	12.7 (0.03)^A^	14.1 (0.69)^A^	19.2 (0.49)^A,^*	20.5 (0.42)^A^	18.6 (0.07)^A^	19.5 (0.52)^A,^*
	En-cont	−2.1 (0.49)	0.21 (0.22)	2.9 (0.1)	–0.06 (0.08)	1.2 (0.01)	11.2 (0.34)
Resin composite blocks (RCB)	Gr	11.0 (0.32)^B,^*	10.8 (0.45)^B^	11.6 (0.76)^B^	10.7 (0.27)^B,^*	11.1 (0.47)^B^	13.0 (0.04)^B^
	Gr-cont	0.9 (0.04)	3.5 (0.26)	6.9 (0.09)	2.8 (0.19)	4.5 (0.29)	9.7 (0.52)
	Lu	5.1 (0.22)^C^	9.0 (0.5)^C^	15.1 (0.72)^C^	11.6 (0.37)^C^	14.9 (0.07)^C^	19.1 (0.59)^A^
	Lu-cont	0.2 (0.39)	0.5 (0.13)	6.0 (0.11)	2.3 (0.39)	4.5 (0.07)	7.9 (0.91)
	Bc	6.1 (2.61)^D^	6.8 (0.03)^D^	6.6 (0.7)^D^	7.8 (0.74)^D^	10.3 (0.53)^D^	12.3(0.57)^B^
	Bc-cont	3.7 (0.36)	3.8 (0.12)	3.7 (0.05)	−0.25 (0.38)	−0.08 (0.99)	3.2(0.91)
	Cs	6.8 (0.69)^D,^*	7.3 (0.07)^E^	6.7 (0.35)^D^	6.3 (0.34)^E,^*	8.5 (0.22)^E^	12 (3.91)^B^
	Cs-cont	−1.0 (0.5)	0.1 (0.59)	0 (0)	0.13 (0.12)	0.25 (0.57)	8.9 (0.13)

Vita™ Enamic (EN); Grandio™ Blocs (GR); Lava™- Ultimate (LU); BRILLIANT™ Crios (BC); Cerasmart™ (CS); Control (cont).

Values with the same superscript letters represent a non-significant difference among different materials (the Tukey post hoc tests, *α* = 0.05), at each time point.

Values with the same superscript asterisk represent a non-significant difference for polished/unpolished surfaces for each material at each time point; the independent sample t test.

There was a significant difference for bleached/unbleached (control) samples for both polished/unpolished surfaces for each material at each time point; the independent sample t test.

After 60 min bleaching of the polished samples, the highest reduction of hardness was found for En, followed by Gr, Cs, Bc and Lu, with significant differences between all materials except Cs and Bc. The same was observed for the unpolished samples in the following order: En, Lu, Gr, Bc and Cs. There were significant differences between all the materials. Notably, there was an insignificant difference in the reduction of hardness between the polished and unpolished Gr and Cs specimens (Table 2).

After 24 h bleaching of the polished samples, the reduction of hardness was the highest for En followed by Gr, Lu, Cs and Bc, with significant differences between all the materials. The same was observed for the unpolished samples in the following order: En, Gr, Lu, Bc, and Cs. A significant difference was observed between all the materials. Whereas, remarkably, there was a significant difference in hardness reduction between the polished and unpolished samples for all investigated materials.

One-month post-bleaching, the reduction of hardness of the polished samples was the highest for En followed by Lu, Gr, Cs and Bc, with a significant difference between all the materials except Cs and Bc. The same was observed for the unpolished specimens in the following order: En, Lu, Gr, Bc and Cs. A significant difference was observed for all the materials except Cs and Bc. Notably, there was a significant difference in the reduction of hardness between the polished and unpolished samples for all the investigated materials except En.

Two-way ANOVA showed a significant material effect and time effect, including a significant interaction for hardness reduction (p < 0.0001) with more influence of the material effect. The greatest influence in the polished group was for the CAD-CAM material (partial eta squared ηp^2^ = 0.798) followed by the interaction effect (ηp^2^ = 0.099) while time had the lowest effect (ηp^2^ = 0.063). The greatest influence in the unpolished group was for the CAD-CAM material (partial eta squared ηp^2^ = 0.84) followed by the time effect (ηp^2^ = 0.11) while interaction had the lowest effect (ηp^2^ = 0.05).

**Figure 3:**
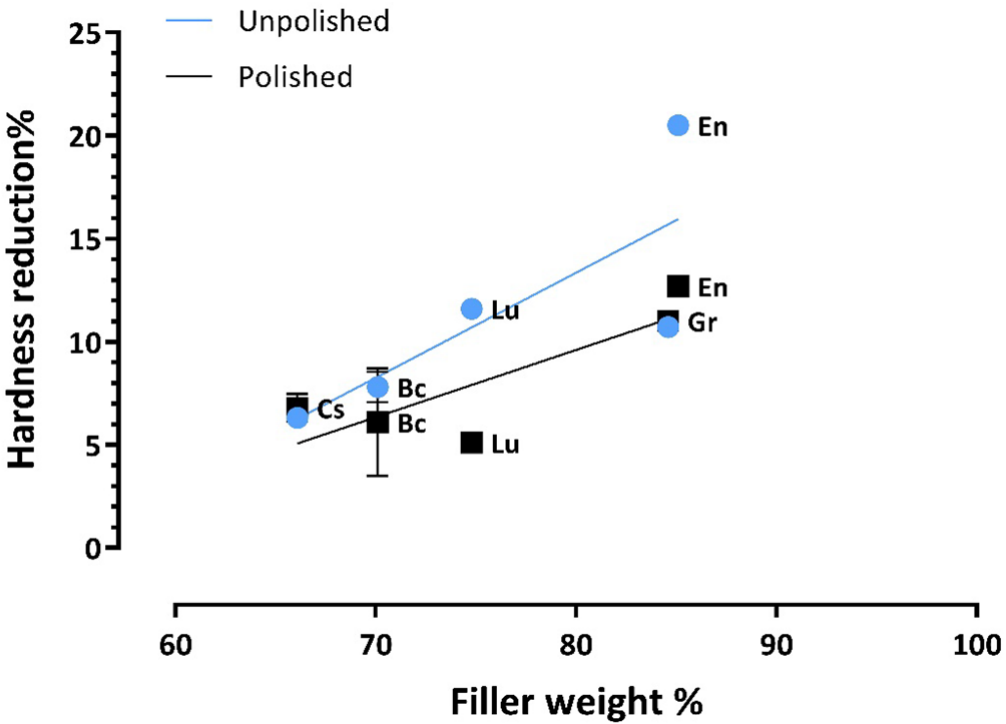
A scatter plot showing an insignificant positive correlation between filler weight (measured experimentally) and hardness reduction in the polished group (R^2^ = 0.7, p = 0.076) and in the unpolished group (R^2^ = 0.62, p = 0.115).

The Pearson correlation of the reduction of hardness and filler weight percentage (data obtained from a previous study^32^ ) showed an insignificant positive correlation between filler weight and the reduction of hardness in the polished group R^2^ = 0.7, p = 0.076 and filler weight and the reduction of hardness in the unpolished group R^2^ = 0.62, p = 0.115. There was also an insignificant positive correlation in the reduction of hardness between polished and unpolished groups R^2^ = 0.52, p = 0.166 (Fig. 3).

**Figure 4:**
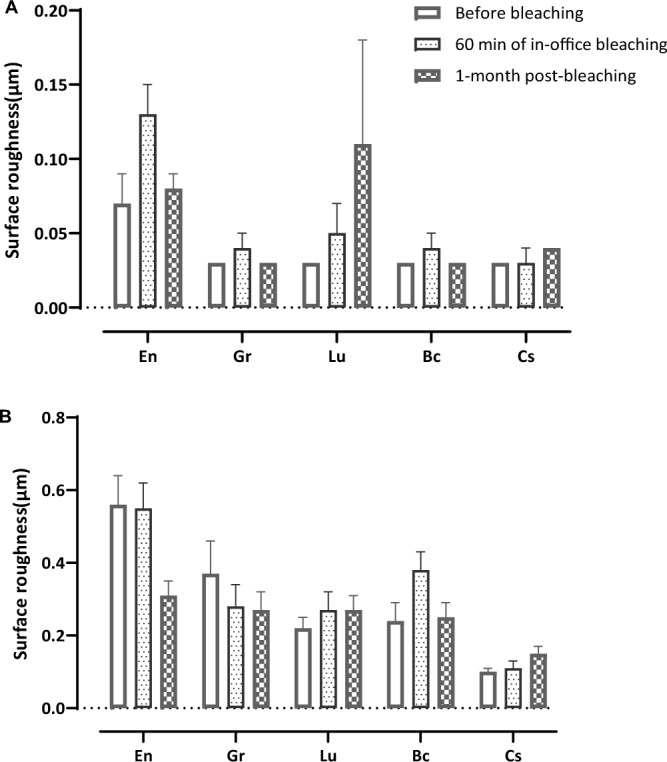
A bar chart illustrating the mean values of surface roughness (Ra) of polished (**A**) and unpolished (**B**) samples before, after 60 min of in-office bleaching and 1-month post-bleaching. Error bars represent the standard deviation.

R_a_ values recorded before bleaching, directly after bleaching, and after one month are presented in Table 3 and Fig. 4. The bleaching treatment did not influence surface roughness for all the investigated materials in the polished group except En and Lu. En showed significantly increased R_a_ values after bleaching from both before bleaching and at one-month post-bleaching. Lu from the polished group exhibited significantly increased R_a_ values after a month. Bleaching influenced the surface roughness of all investigated materials in the unpolished group. En showed significantly reduced R_a_ values after one-month post-bleaching from both before and after bleaching. Also Gr showed significantly reduced R_a_ values after 60 min of bleaching and one month post bleaching from that of before bleaching. Lu showed significantly increased R_a_ values after one-month post-bleaching and after 60 min of bleaching from that before bleaching. Cs showed significantly increased R_a_ values after bleaching from both before bleaching and one-month post-bleaching. Finally, Bc showed significantly increased R_a_ values after one month of bleaching compared to both before and after 60 min of bleaching.

**Table 3:** The mean and standard deviation values of surface roughness (R_a_) of polished and unpolished samples before bleaching, after 60 min of in-office bleaching and one-month post-bleaching**.**

Materials		Surface roughness (µm) of polished samples; mean (SD)			Surface roughness (µm) of unpolished samples; mean (SD)		
		Before bleaching	After 60 min bleaching	After 1-month post-bleaching	Before bleaching	After 60 min bleaching	After 1-month post-bleaching
PICN	En	0.07 (0.02)	0.13** (0.02)	0.08 (0.01)	0.56 (0.08)	0.55 (0.07)	0.31** (0.04)
Resin composite blocks (RCB)	Gr	0.03 (0.00)	0.03 (0.01)	0.04 (0.00)	0.37** (0.09)	0.28 (0.06)	0.26 (0.05)
	Lu	0.03 (0.00)	0.05 (0.02)	0.11** (0.07)	0.22** (0.03)	0.27 (0.05)	0.27 (0.04)
	Bc	0.03 (0.00)	0.04 (0.01)	0.03 (0.00)	0.24 (0.05)	0.38** (0.05)	0.25 (0.04)
	Cs	0.03 (0.00)	0.03 (0.01)	0.04 (0.00)	0.10 (0.01)	0.11 (0.02)	0.15** (0.02)

Values with superscript asterisks represent significant difference from other values for each material at each time point in each group (polished and unpolished), one way ANOVA.

**Figure 5:**
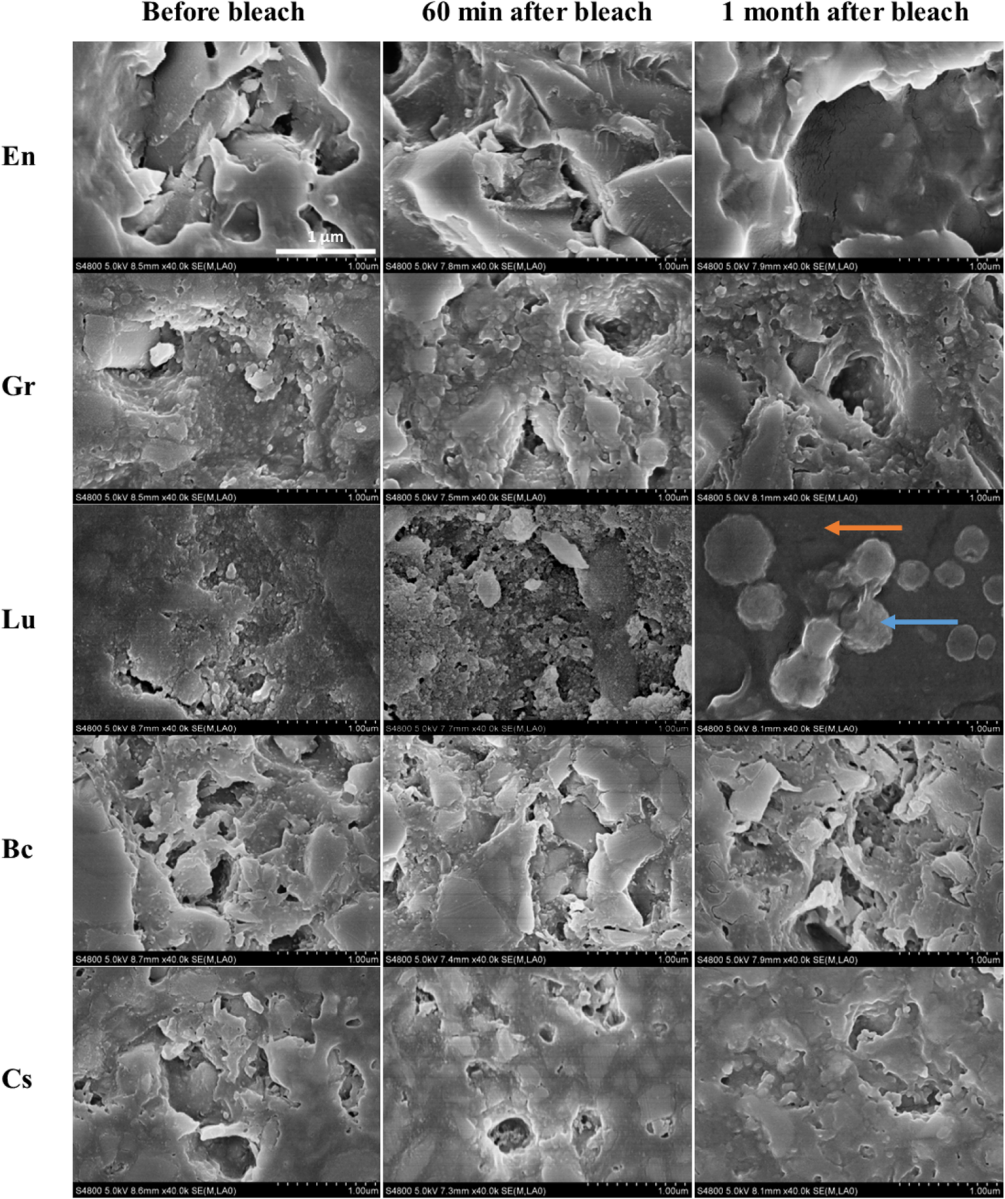
SEM images at ×40k of the polished surfaces of all investigated materials before bleaching, 60 min after bleach, after a month from that of unbleached condition. Note the less demarcated particles margins and more rounded particles after bleach, and after a month from that of unbleached condition. Lu showed more dark spaces (polymer); orange arrows showed more prominent clustering of filler particles (blue arrows).

**Figure 6:**
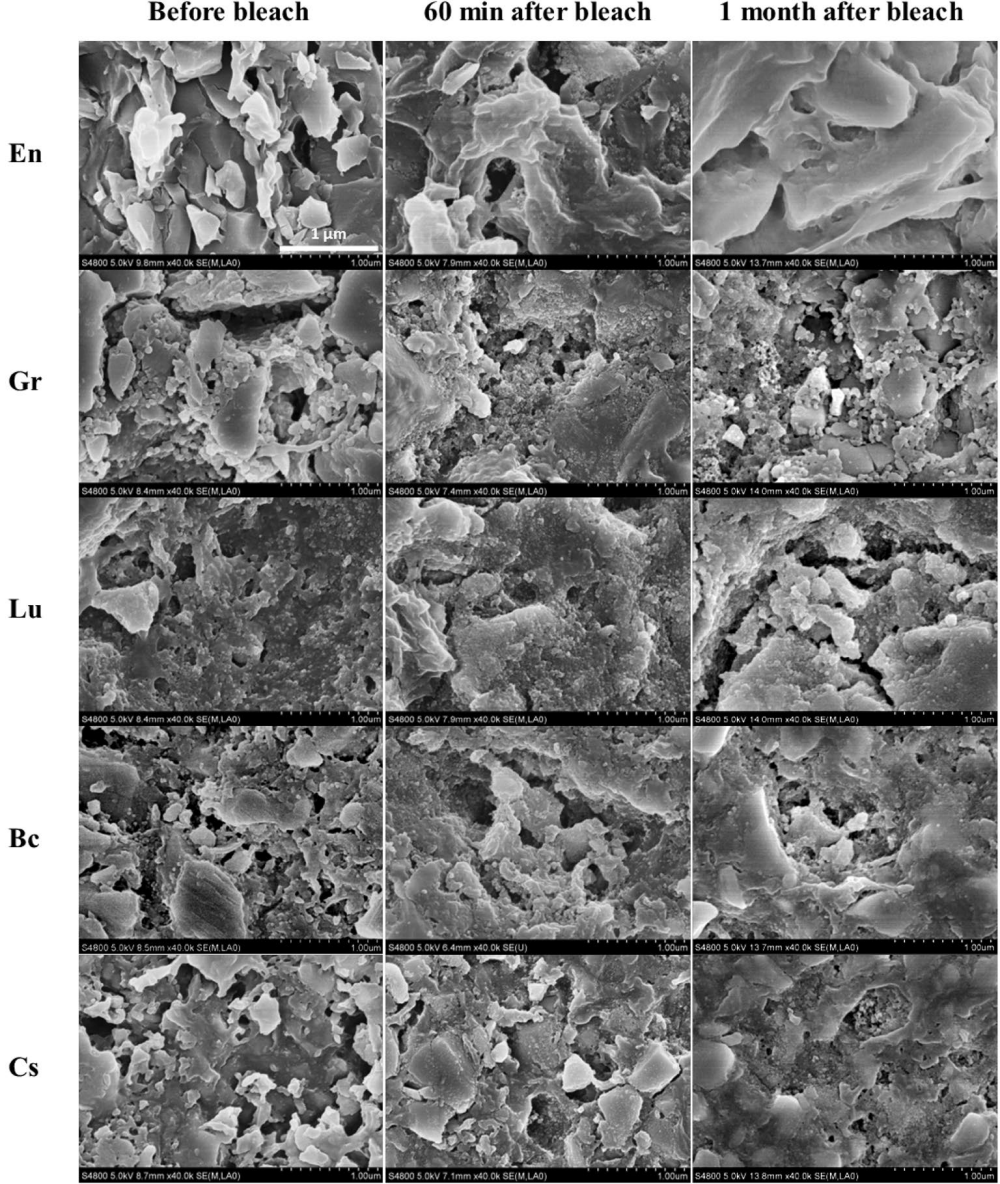
SEM images at ×40k of the unpolished surfaces of all investigated materials before bleach, 60 min after bleach, and after a month from that of unbleached condition. Note the less demarcated particles margins and more rounded particles after bleach and after a month from that of unbleached condition.

SEM images at a × 40k magnification showed less demarcated particle margins and more rounded particles directly after bleaching and after one-month post-bleaching when compared with both the polished and unpolished samples (Figs. 5 and 6). Small cracks were found in the matrix phase of both polished and unpolished specimens, especially one-month post-bleaching. For the polished specimens, En and Gr showed deeper grooves, whereas Bc and Cs exhibited no remarkable changes after one month. Nevertheless, Lu presented more dark spaces (the polymer matrix) and more prominent clustering of filler particles. More remarkable surface deterioration and margin fractures were observed for the unpolished specimens than for the specimens in the polished group. Unpolished Cs and En did not exhibit remarkable surface morphology variations.

## Discussion

In the present study there was a significant difference in hardness reduction percentages between the bleached and unbleached (control) specimens for each material, and thus, the first null hypothesis was rejected. Moreover, there were significant material and time effects, with a significant interaction for the reduction of hardness (p < 0.0001) with more influence from the material effect. Given that, the second null hypothesis was rejected as well. Markedly, there was a significant difference in the reduction of hardness between polished and unpolished specimens for all investigated materials, and thus, the third null hypothesis was rejected. The filler weight percentage showed an insignificant effect on the reduction of hardness, and thus, the fourth null hypothesis was accepted. The bleaching influenced the surface roughness of some of the investigated materials, and thus, the fifth null hypothesis was partially accepted.

En showed the highest hardness reduction of all investigated materials for both the polished and unpolished surfaces at all time points. This was an interesting finding as En is a PICN composed of an interpenetrating ceramic-polymer network that is strong and resistant to breakdown^41^. Previous studies have found that En has the least hardness reduction when compared to other RCBs upon storage in different media^42,43^. However, this contradicted the findings of some alternative studies, which, in contrary, showed an increase in the material microhardness^44,45^. This might be attributable to the fact that bleaching agents can penetrate and diffuse through the polymeric matrix of the material to a greater extent than the other storage media such as water, artificial saliva or 70% ethanol solution^43^ due to oxidative cleavage of the polymer chains^46^ or similar solubility factor to that of the bleaching agent^47^. Furthermore, the samples in this study were stored in water for three months to simulate clinical conditions as such the materials would have been bleaching after servicing for a while in the oral cavity while the compared studies have measured bleaching effect without any previous storage in water.

Nevertheless, RCBs were less affected by the in-office bleaching procedure. GR exhibited the highest reduction of hardness of all the resin-composite blocks. However, it has the highest filler percentage (86 wt.%). This, again, contradicted previous findings^42,48^ and could be attributed to the aforementioned reason of different experimental conditions. Lu, Bc and Cs had similar filler weight percentages (75 wt.%, 70 wt.% and 66 wt.%) and the same level of the reduction of hardness. Lu contains more zirconium silicate in its filler composition; as such, it is more prone to hydrolysis of the silane-coupling agent as a consequence of inefficient salinization of high crystalline content in the zirconium silicate^49^or due to material inhomogeneity and presences of large filler particles^50^. Then again, silicate materials are more prone for silane-aided adhesion promotion than zirconia or zirconium silicate. However, silanes may degrade over time in resin composites^51^. Furthermore, nano-cluster filler particles in Lu typically have defects and voids, which could explain the higher levels of surface degradation^4^. Lu with zirconia nanoparticles has been found to exhibit higher softening resistance compared to En with a feldspathic ceramic structure. This is in line with studies reporting that zirconia-containing materials have higher hardness and strength values than feldspathic-containing materials^52,53^. Although bleaching agents can influence the surface microhardness of dental restorations, the mechanism for such changes is not fully clear. One possible explanation is that the peroxides in bleaching agents release very reactive free radicals at the resin-filler interphase. These free radicals may attack glass particles, silica, and alumina, causing a separation of fillers and filler-matrix debonding^54^. Alternative explanations are that the bleaching agents can cause an oxidative cleavage of the polymer chains^46^ or that the resin matrix has a similar solubility factor to that of the bleaching agent^47^. Bleaching agents might have a greater effect on the surface microhardness at higher temperatures^28^.

All unpolished surfaces exhibited a significantly higher hardness reduction. When servicing in the oral environment and exposed to fluids, acids and moisture, the surface roughness of the materials tends to increase^9,30–32^, rendering the restoration more prone to penetration by bleaching agents which affect their physical properties, such as microhardness^35,55^. Our finding might indicate that polishing dental restoration before bleaching procedures is advisable to reduce any potential softening of the restorative materials.

Two-way ANOVA showed significant material and time effects with a significant interaction for hardness reduction (p < 0.0001) with more influence from the material effect. Considering the different resin matrices and filler compositions of CAD/CAM composite blocks, the bleaching agents can cause various changes in the material’s microhardness and surface roughness. This indicates that bleaching effects are material dependent.

The reduction of hardness was greatest after 60 min bleaching and increased slightly after 24 h and after one month. This finding is consistent with other studies, as most material changes occur in the first interaction with a solvent or contacting solution, here a bleaching agent, after which the material effect may reach an equilibrium^56–59^. Further, the bleaching agent might be diluted with a storage medium (deionized water) after performing the bleaching for the recommended time. In this study, the lowered hardness values were positively correlated with the amount of filler loading for all CAD/CAM composite materials, where En with the highest weight percentage of fillers (86%), exhibited the least softening resistance. On the other hand, factors other than filler type and concentration, such as microstructure homogeneity, cross-linking between the fillers, and resin matrix, have a significant impact on the interaction between the bleaching agents and dental materials^54^. However, all the materials experienced hardness reduction over time due to plasticization of the polymer matrix caused by water sorption and consequently degradation and reduction in material stiffness^42,60^.

In this research, both profilometry and SEM were used to study the surface topography of the materials of interest. In-office bleaching did not influence the surface roughness of any of the investigated materials of the polished group except for En, which showed significantly increased R_a_ values after 60 min bleaching when compared with the values before bleaching and one-month post-bleaching. Polished Lu specimens exhibited significantly increased R_a_ values after one month. This finding was in line with similar studies^29,54^. This can be attributed to the Bis-GMA composition of Lu, as Bis-GMA-containing materials have higher surface roughness values^46^. Furthermore, for the unpolished group, the lower R_a_ values of Lu might be attributed to the fact that it has smaller filler size than En or the highly cross-linked polymer matrix and zirconia fillers of Lu, which resist free radicals caused by bleaching agents^54^.

Furthermore, the bleaching agent and diverse dental materials may chemically interact in a way that changes the surface topography^54^. Surface roughness might increase due to the composition, exposure time, and concentration of bleaching agents^61^. The increased surface roughness of dental restorations can cause discolouration and secondary caries by enhancing and accelerating biofilm accumulation. The R_a_ value should not exceed 0.2 μm to prevent biofilm accumulation^62^. This value had not been exceeded for all polished samples; however, it was exceeded for all the unpolished samples except CS. Bleaching influenced the surface roughness of for all the investigated materials in the unpolished group of specimens.

Compared to the unbleached condition, SEM images at ×40k for the polished specimens, En and Gr exhibited deeper flaws, whereas Bc and Cs showed no significant changes after one month. Lu contained more dark spaces (the polymer matrix) and a more prominent clustering of filler particles. That being said, more remarkable surface deterioration and margin fractures were observed on the unpolished specimens compared with the polished group. Unpolished Cs and En did not present significant surface morphology variations.

In this study, all samples were stored in water at 37 °C as simulator of intraoral fluids^61^ and temperature and being more reflective of the clinical conditions. According to ISO standards (ISO 10993-13), any devices intended to be used for more than 30 days and tested in simulated conditions should be tested at 1-month, 3-month, 6-month, and 1-year^40^, hence a short term storage for 3 months was applied.

Future work should involve bleaching at high temperatures and using 3D micro-CT or AFM to facilitate a deeper understanding of the changes bleaching causes inside the materials.

## Conclusion

The following can be concluded: the hardness of CAD/CAM composite blocks was affected by in-office bleaching, with PICN exhibiting the least hardness stability of all of the resin-composite blocks. In-office bleaching significantly influenced the surface roughness of unpolished CAD/CAM composite blocks. In summary, polishing the restorations in advance of a bleaching procedure is advisable.

## Data Availability

The datasets used and/or analysed during the current study are available from the corresponding author on reasonable request.
